# Local autograft versus mixture of autograft and allograft combination with posterior instrumentation for adolescent idiopathic scoliosis: A retrospective comparative clinical study

**DOI:** 10.1097/MD.0000000000042443

**Published:** 2025-07-25

**Authors:** Mehmet Nuri Erdem, Yigit Kultur, Abdulhalim Akar, Mehmet Aydogan

**Affiliations:** a Orthopedics and Traumatology, Yeni Yuzyil University Gaziosmanpasa Hospital, Istanbul, Turkey; b Department of Spine Surgery, Memorial Sisli Hospital, Istanbul, Turkey.

**Keywords:** allografts, autografts, scoliosis

## Abstract

Adolescent idiopathic scoliosis (AIS) is a common spinal deformity usually treated with a surgical procedure comprising spinal fusion. Solid fusion after such surgery is important for the overall success of the procedure, where graft selection is also a significant factor. Allografts are as effective as autografts in the adolescent patient population; however, a major limitation is their high cost. The purpose of this investigation was to reassess the necessity for allografts when surgically treating AIS and compare the results between the use of only local autograft and a combination of autograft and allograft. Fifty-four patients with AIS with minimum follow-up of 2 years were assigned to 2 groups and analyzed. In 28 patients with a mean age of 14.2 years, local autografts harvested from the thoracic and lumbar vertebrae facet joints were used (autograft group). Over the top and bottom 3 segments of the fusion site, grafts were placed. Various combinations of autografts and allografts were applied over all fusion levels in 26 patients with a mean age of 15.1 years (allograft group). The mean follow-up period was 45.2 months in the autograft and 45.7 months in the allograft group. Mean 59.8 cc of freeze-dried crushed cancellous graft was used in the allograft group. The surgical outcomes were compared between the autograft and allograft groups. The mean of the major curvature angle is preoperatively measured as (59.9°, 63.9°); early postoperatively (11.5°, 13.8°) and (16.5°, 18.5°) at the final follow-up visit. Curve correction was calculated early postoperatively as 81.2%, 79.4%, 73.0%, and 72.3% at the last follow-up, respectively. All patients in both groups attained fusion. The major disadvantages in the use of allografts these days is the increased surgical cost. Our grafting technique thus demonstrated that allografts are not necessary as local autografts when used correctly would achieve fusion.

## 1. Introduction

Surgical treatment of adolescent idiopathic scoliosis (AIS) aims to correct the deformity and to create adequate bony fusion to maintain the correction. The use of autogenous and/or allogeneic grafts is a standard procedure to increase the rate of posterior arthrodesis. Locally harvested autografts, such as the iliac crest and ribs, can be used in spinal surgeries.^[1–5]^ Allografts have demonstrated success in adolescent thoracolumbar spines almost comparable with those of autografts.^[6–10]^ The major limitations that are placed on the use of allografts are those involving adverse effects that it places on surgical costs. Several authors have preferred using locally harvested autografts alone to obtain fusion in an attempt to avoid both the disadvantages of allografts and morbidity caused by the use of iliac bone grafts.^[6,11–13]^ However, spinous processes have to be used to obtain a sufficient amount of graft in such procedures, which in turn leads to cosmetic defects.

For the patients in the present series, the thoracic vertebrae facet joints and the transverse processes served instead of the spinous processes as a source for the local autograft. The present study aimed to compare the clinical and radiological results of AIS patients in whom local autografts were applied only at the proximal and distal fusion levels with those in whom autografts and allografts were applied together at the fusion site in their surgical treatment with posterior segmental instrumentation. Herein, the authors aimed to describe the grafting technique in a patient group where fusion was achieved using autografts.

## 2. Methods

### 2.1. Study design and patient selection

Patients who were diagnosed with AIS and planned to undergo surgical treatment with posterior instrumentation and fusion between January 2018 and December 2021 were evaluated retrospectively. The study obtained its approval from the local ethics committee of the Istanbul Yeni Yuzyil University Clinical Research Ethics Committee (Number: 01-1433). Written informed consent was obtained from all patients and their parents for publication of their cases with accompanying images. Patients who were diagnosed with AIS, had a Cobb angle of >40°, were 20 years of age or younger, planned to undergo primary posterior instrumentation, and had a Risser score of 3 or more were included in the study. Patients with additional surgical interventions other than posterior spinal instrumentation, such as anterior release or osteotomy, were excluded because these interventions would have an impact on the fusion rate. As the use of local autografts alone was planned, patients who had undergone thoracoplasty were excluded. In addition, patients with a Risser score of 1 and 2 were also left out as they might have developed curvature progression related to growth potential, such as “the phenomenon of the crankshaft,” despite solid fusion. Data such as age, sex, weight, and height before surgery and body mass index (BMI) were noted in all included patients.

All surgeries were performed by 2 senior authors (MA and MNE), who are expert surgeons in spinal deformity. Both surgeons performed the surgeries at the same institution. An index surgeon performed the grafting technique. In the surgeries performed by MA, fusion was performed using only local autografts (autograft group) [n = 30]. In surgeries performed using MNE, fusion was aimed at using a combination of allografts and autografts (allograft group) [n = 29]. Each senior surgeon applied his technique to all patients, so there was no randomization in either group. Fusion was evaluated using X-ray, looking at the absence of radiolucent lines around the implants, the trabecular bone continuity across the grafted region, and the absence of pseudarthrosis or hardware failure as seen in follow-up radiographs. Patients meeting the inclusion criteria at the beginning of the study were assigned to one of these 2 groups and later underwent surgery, regardless of the radiological or demographic characteristics.

### 2.2. Radiological studies

Straight and bending anteroposterior and lateral radiographs of the entire spinal column were obtained preoperatively. All the patients underwent magnetic resonance imaging of the cervical, thoracic, and lumbar regions in order to ensure that intramedullary pathologies were excluded. Cobb angles of the curvatures were measured on anteroposterior radiographs and Risser scoring was performed. Flexibility of the curvatures was measured using supine bending radiographs. All curvatures were classified according to the Lenke classification.^[14]^ The levels of fusion were determined by senior authors according to the outcomes of the above inquiries. Radiographs were obtained at postoperative 6th week, 1st year and 2nd year.

### 2.3. Complications and outcome evaluation

All complications were recorded both intraoperatively and during the follow-up period. Loss of correction, fusion defect, implant loosening or breakage, and the presence of pseudarthrosis were evaluated on postoperative radiographs. All patients who complained of persistent severe back pain, defect, unfused facet in the fusion mass, or loss of correction of more than 10° when compared with the early postoperative radiographs were prediagnosed with pseudarthrosis and then evaluated with computed tomography (CT) scans.^[15,16]^

### 2.4. Surgical technique

**Figure 1.**
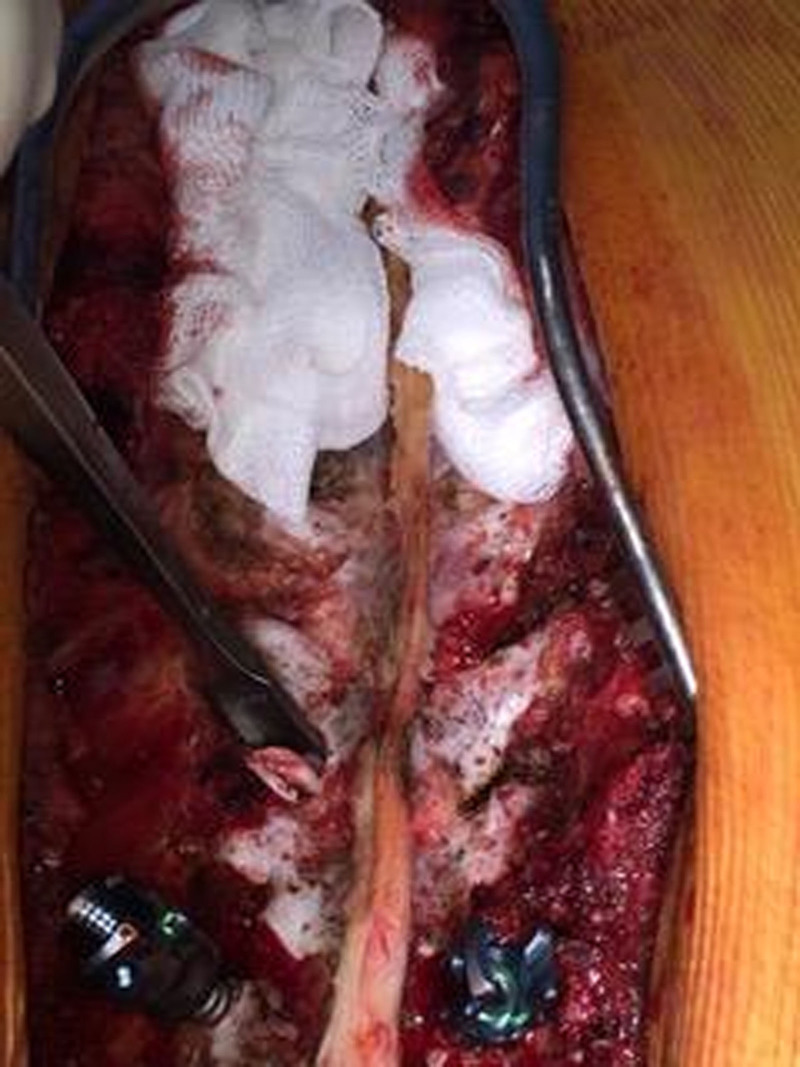
Intraoperative photo shows operation field. Note excision of the facets using a gouge osteotome. To facilitate fusion all soft tissues upon the bones were removed.

The same surgical technique was employed for all patients in both groups. A gouge osteotome was used for the excision of the facet joints, and the harvested bones were used later as grafts (Fig. 1). The cartilage on the facet joint surfaces was removed using an osteotome or a high-speed drill. When the paraspinal muscles are dissected bilaterally, attention is paid to remove all soft tissues using a Cobb elevator. The pedicle screws (Medtronic Inc., Memphis, TN) were bilaterally placed on all levels that were planned to correct, and fusion was performed. At the thoracic level, the posterolateral bony parts of the transverse processes were partially excised using a rongeur. The excised bony parts were retained for grafting. After kypholordotic angling of the rods, which were placed later, correction of the deformity was performed. The harvested bone grafts were stripped off their soft tissues and washed with a saline solution. The volume of the harvested grafts was measured using two 20 cc syringes. First, the grafts were filled in a syringe, and 20 cc of saline solution from the second syringe was injected into the first syringe with the grafts until the volume of the graft syringe reached 20 cc. The volume of saline solution left in the saline syringe was recorded as the volume of the bone graft. The volume of the allografts was measured using the same technique before they were placed in the surgical field.

**Figure 2.**
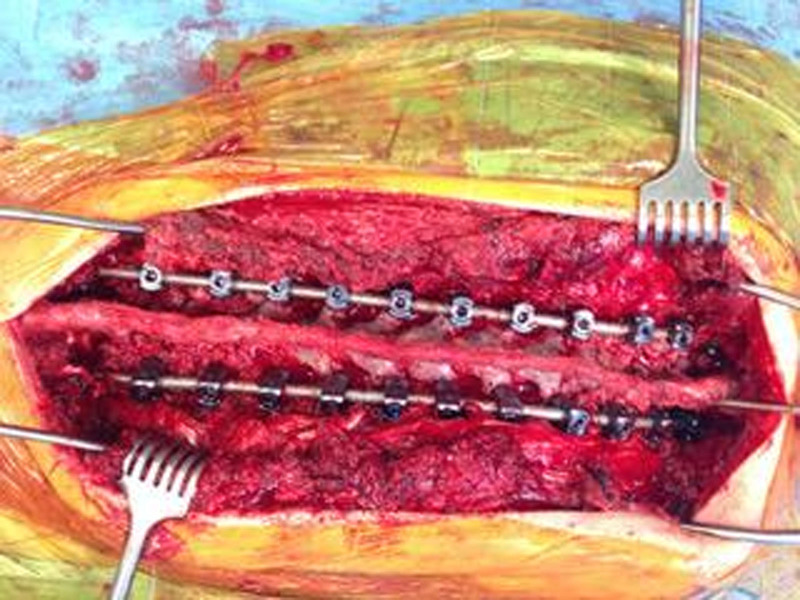
Placement of the locally harvested autografts in the proximal and distal 3 levels of the instrumentation site. Grafting procedure is not applied on the rest segments.

**Figure 3.**
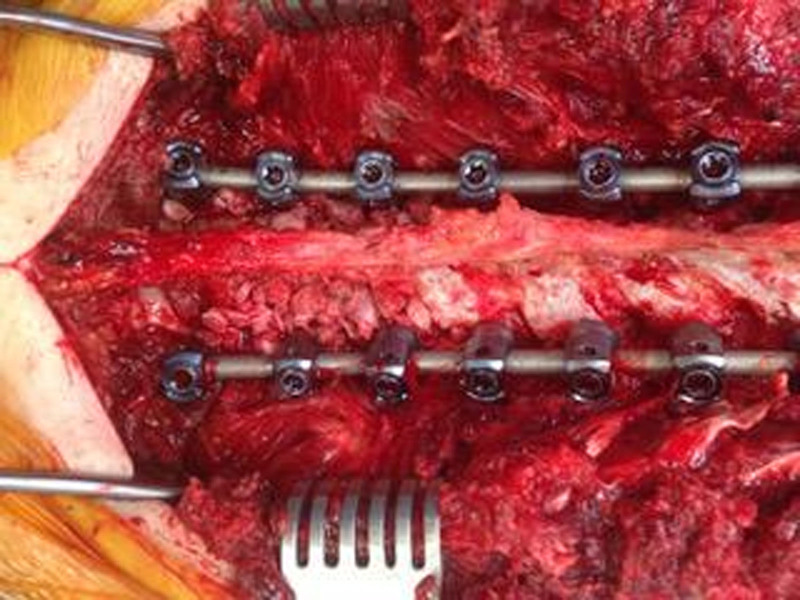
Autografts mixed with 5 cc for each level of freeze-dried cancellous crushed allografts (AlloSource, Centennial, CO) then the mixture is placed on all facet joints and laminas of the whole fusion site in the allograft group.

The locally harvested autografts in the patients of the autograft group were placed on the facet joints and laminae on the top and bottom 3 vertebrae. The grafting procedure was not applied to the remaining segments (Fig. 2). However, autografts were mixed with 5 cc for each level of freeze-dried cancellous crushed allografts (AlloSource, Centennial, CO), and the mixture was placed on all facet joints and laminae of the entire fusion site in the allograft group (Fig. 3). Sterile allografts were used and washed using saline solution only, without re-sterilization.

### 2.5. Statistical analyses

IBM SPSS 22.0 software is utilized for statistical analysis. Descriptive statistics that were used to evaluate the study data include frequency and percentage distribution, mean, median, and standard deviation. For intergroup comparisons, *X*^2^ (chi-square) analysis will be performed. Intergroup comparisons of the normally distributed parameters with the quantitative data were performed using the *t* test. Correlation analysis was done in order to find the relation between the amount of locally harvested autografts and BMI. All of the analyses were performed at a level of significance of α = 0.05.

## 3. Results

Fifty-nine patients who met the inclusion criteria were enrolled for this study. The study was completed by 54 patients (91%) whose clinical and radiological data for at least a follow-up period of 2 years were available. The data of 4 patients from other countries and 1 patient lost to follow-up were unavailable. The study sample consisted of 28 of 30 patients (93%) from the autograft group and 26 of 29 patients (89%) from the allograft group.

The mean age of the autograft group (23 females and 5 males) is 14.2 (12.1–18.1) years, while that of the allograft group (22 females and 4 males) is 15.1 (11.9–19.8) years. The mean follow-up periods were 45.2 (28–67) and 45.7 (29–67) months, respectively. The mean BMI of both groups are 21.0 (15.7–31.6) and 21.2 (16.0–30.9), respectively. There is no significant difference in the age, follow-up period, and body mass index of both groups (Table 1). The curve types were classified according to the Lenke classification (Table 2).

**Table 1:** Patients characteristics.

	Autograft group (n = 28)	Allograft group (n = 26)	*P*; OR*
Age (years) (SD; range)	14.2 (1.58; 12.1–18.1)	15.1 (1.80; 11.9–19.8)	.87
Follow-up (months)	45.2 (13.9; 28–67)	45.7 (13.2; 29–67)	.96
Female	23	22	.55; 1.2
Male	5	4	.55; 0.84
BMI	21.0 (4.2; 15.7–31.6)	21.2 (3.6; 16.0–30.9)	.97

BMI = body mass index.

* OR = odd ratio.

**Table 2:** Curve types of 2 groups according to the Lenke classification system.

	Autograft group (n = 28)				Allograft group (n = 26)			
		Lumbar modifier				Lumbar modifier		
		A	B	C		A	B	C
1	14	7	6	1	11	5	4	2
2	3	1	2		10	3	7	
3	4			4	2			2
4					1		1	
5								
6	7			7	2			2

Using Cobb method, the mean of the major curvature angle of the autograft group is preoperatively measured as 59.9 (43–80) degrees; while it is early postoperatively measured as 11.5 (0–25) degrees and 16.5 (6–27) degrees at the final follow-up visit. However, that of the allograft group is preoperatively measured as 63.9 (41–82) degrees; while it is early postoperatively measured as 13.8 (0–29) degrees and 18.5 (0–31) degrees, respectively, at the final follow-up visit. Curve correction in the autograft group was calculated as 81.2% (64.2%–100%) on average and 73.0% (61.2%–87.7%) at the last follow-up, while it was calculated as 79.4% (62.8%–100%) on average and 72.3% (57.1%–90.3%) at the final follow-up visit for the allograft group. In the autograft group, the mean loss of correction was 5.0 (0–10) degrees on average and 4.7 (0–12) in the allograft group. Thus, the radiological parameters were not significantly different between the groups (Table 3). Instrumentation was performed on an average of 11.8 (8–15) levels in the autograft and 11.8 (9–15) levels in the allograft group (Table 4).

**Table 3:** Radiographic measurements of 2 groups.

	Autograft (n = 28)	Allograft (n = 26)	*P*
Cobb angle major curve (°)			
Preoperative mean (SD; range)	59.9 (9.5; 43–80)	63.9 (11.5; 41–82)	.17
Postoperative	11.5 (7.5; 0–25)	13.8 (7.6; 0–29)	.07
Follow-up	16.5 (16; 6–27)	18.5 (17; 0–31)	.89
Curve correction (%)			
Postoperative	81.2 (7.2; 64.2–100)	79.4 (8.7; 62.8–100)	.15
Follow-up	73.0 (7.3; 61.2–87.7)	72.3 (10.6; 57.1–90.3)	.91
Loss of correction (°)	5.0 (2.6; 0–10)	4.7 (2.5; 0–12)	.90

**Table 4:** Instrumentation levels of 2 groups.

	Autograft group			Allograft group		
	T2	T3	T4	T2	T3	T4
T10				1		
T11	1		2			
T12	2		4	6		
L1	4			6		1
L2	4			4		1
L3	2	1	1	1		3
L4	1	1	5			3

An average of 5.1 (4–7) cc of autograft was locally harvested in the autograft group, while that of the allograft group was 5.6 (4–7) cc (Table 5). Accordingly, grafts were set on the proximal and distal segments in the autograft group. A mean of 0.85 cc of local autograft was used for each level. However, the autografts are placed on the whole surgical field in the patients of the allograft group, an average of 0.47 cc of local autograft is placed on each level. No significant relationship was detected between the amount of harvested autograft and BMI in the analysis (*R* = 0.211, *P* = .12).

**Table 5:** Comparison of the 2 groups in terms of the locally harvested graft volume, the patients scanned with computed tomography (CT) due to prediagnosis of pseudarthrosis and the patients who developed deep infection.

	Autograft (n = 28)	Allograft (n = 26)	*P*
Graft volume (SD; range)	5.1 (2.9; 4–7)	5.6 (3.7; 4–7)	.15
CT	2 (7.1%)	2 (7.7%)	.65
Infection	0	1 (3.84%)	.48

CT = computed tomography.

An average of 59.8 cc (5.03 cc per level) of allogeneic chip graft was used in the allograft group. When the same volume measurement technique for the autografts was applied in the measurement of allogeneic chip grafts, the mean volume of 30 cc freeze-dried cancellous allograft in 10 measurements was 5.1 cc. According to our measurement technique, a 0.8 cc allograft, in addition to an autograft, was placed on each level in this group.

Postoperative radiographs taken at the second year follow-up revealed loss of correction of >10° in Cobb angle of major curvature in 1 patient from the autograft group and 2 patients from the allograft group. The CT scans of all 3 patients showed solid fusion. A CT scan was performed to investigate 1 patient from the autograft group who experienced persistent back pain, but no findings of pseudarthrosis were detected.

Deep infection was observed in 1 patient from the allograft group who was reoperated on the postoperative 18th day. In this patient, all grafts were removed, followed by 2 debridement operations that resulted in healing and solid fusion. For infection, the difference was not significant between the 2 groups (Table 5).

## 4. Discussion

Graft application to increase the fusion rate has been a common practice in surgical treatments of AIS. Autogenous and allogeneic biological grafts and synthetic grafts were employed for this purpose, and the benefits and disadvantages of all were debated in many series. Facet joints, laminas, and spinous and transverse processes can be used as local autograft sources from posterior spinal instrumentation.^[1,3,5,17]^ Besides these, the iliac crest and ribs can be used as autografts with additional exposure.^[4,10]^

Graft harvesting from the iliac crest may be associated with numerous morbidities including increased bleeding, extended surgical time, neurological injury, gait disturbance, fractures, painful scars, and cosmetic defects.^[18–20]^ The most common complication encountered after surgery is chronic donor site pain with the prevalence amounting to 31%.^[21]^ Grafts of ribs origin have also been used as autografts in patients undergoing thoracoplasties.^[4]^ However, many reports demonstrated a significant reduction in the percentage of the predicted value of pulmonary function tests averaging 28% after the surgery of rib resection.^[14,22]^ The removal of spinous processes may result in cosmetic defects.

Many authors have suggested the use of allografts as an appropriate alternative to autografts.^[6–10]^ However, allografts are osteoconductive. They are produced in different shapes and sizes, have a variety of preparation techniques, and carry a risk of disease transmission.^[11,23–27]^ In the developing countries, usage of the allografts is raising the cost of such operations. Our findings supported that locally harvested autografts are a cost-efficient alternative for the surgical treatment of AIS that obviates the need for the procurement of allograft.

Autogenous and allogeneic grafts have been used together for a long time by 2 senior authors at our institution. The idea of the current study is that spontaneous fusion is observed after graft removal during debridement in patients who experienced early deep infection after posterior instrumentation. This incident raises the question of whether fusion can be achieved without grafting or not. Betz et al^[12]^ reported that similar fusion rates were obtained in AIS patients who were treated using posterior multi-segmented hooks when compared with the no-graft group and the allograft group. In a study by Betz et al^[12]^ study, the authors decided to obtain fusion using local autografts without allografts. The transverse processes of the thoracic vertebrae and facet joints of the vertebrae that underwent fusion were used as autogenous grafts in all the patients. The amount of grafts harvested would not be sufficient for all fusion levels; the grafts were placed on the proximal and distal segments in the autograft group. The allograft group was the control group, and a mixture of auto- and allografts was placed on the entire surgical field.

To assess fusion on X-ray, we focused on the absence of radiolucent lines, the continuity of the trabecular bone, and the nonexistence of signs indicative of implant failure or pseudarthrosis. Pseudarthrosis develops mostly in the thoracolumbar junction and distal to the fusion mass following posterior surgery for AIS.^[28]^ Biomechanically perspective observations indicate that the proximal and distal parts of the screw-rod construct are most exposed to mechanical stress. The grafts were placed into the proximal and distal segments of each fusion level of the autograft group. No grafts were used at any of the remaining levels. Following excision of the facets, the periostea of the posterior components in this region were stripped off using a Cobb elevator. We believe that the rigid fixation achieved through the placement of bilateral pedicle screws at each level helped the occurrence of fusion in this part. The success fusion rates obtained in the allograft group led us to ask that: “Could the fusion be achieved without using auto- or allografts?” However, the risk of developing pseudarthrosis prevents us from applying this surgically; thus, the authors cannot create a control group for ethical issues.

Volumetric measurement of the harvested autogenous grafts was performed as a routine practice in our clinic in another study. The same technique was employed to measure the volumes of local autografts and allografts harvested in this study. Although the amount of graft harvested may vary widely depending on the surgical technique performed, the amount harvested from the facet joints and transverse processes can be considered moderate for posterior surgery of AIS. When applying grafts using the conventional approach at all fusion levels, the amount is not sufficient. However, when the grafts were placed on the proximal and distal segments, adequate grafting was achieved at these levels.

Multiple factors go into the cost of the posterior surgical intervention for AIS; by far, the leading ones included implants (29%), intensive care unit and inpatient room costs (22%), operating room (9.9%), and bone grafts (6%).^[29]^ In our institution, except for graft usage, all operation conditions are the same. Thus, the costs in both groups were assumed to be the same. An average of 59.8 cc (5.03 cc per level) of allogeneic chip grafts was used in the allograft group. This implies an additional cost of USD 2000 for each patient in our country. The only disadvantage of allograft use in our series was the increased surgical cost.

The present study had 3 major limitations. First, 2 different surgeons performing surgery may actually cause technical variations. The 2 surgeons, however, followed institutional policy on procedures of scoliosis correction as outlined by policy guidelines. Second, to assess fusion, CT scans could not be used routinely (for ethical issues such as high-dose radiation is a major cause of some types of cancers). Fusion was assessed using X-rays. Third, based on the retrospective nature of the current study, it was limited by how many patients met with the inclusion criteria over the course of study duration. Future prospective studies must be performed with larger samples to validate these findings.

## 5. Conclusion

Major drawback that weigh against the use of allografts today is the increased cost of surgery. Our grafting technique of fusion using only the application of local autograft without the use of allografts was shown to be feasible. Hence, appropriate surgical procedures can reduce surgical costs.

## Author contributions

**Conceptualization:** Mehmet Nuri Erdem, Mehmet Aydogan.

**Data curation:** Mehmet Nuri Erdem, Yigit Kultur, Abdulhalim Akar, Mehmet Aydogan.

**Formal analysis:** Mehmet Nuri Erdem, Yigit Kultur, Abdulhalim Akar, Mehmet Aydogan.

**Investigation:** Mehmet Nuri Erdem, Mehmet Aydogan.

**Methodology:** Mehmet Nuri Erdem, Mehmet Aydogan.

**Project administration:** Mehmet Nuri Erdem, Yigit Kultur, Abdulhalim Akar, Mehmet Aydogan.

**Resources:** Mehmet Nuri Erdem, Mehmet Aydogan.

**Software:** Mehmet Nuri Erdem, Yigit Kultur, Abdulhalim Akar, Mehmet Aydogan.

**Supervision:** Mehmet Nuri Erdem, Mehmet Aydogan.

**Validation:** Mehmet Nuri Erdem, Yigit Kultur, Mehmet Aydogan.

**Visualization:** Mehmet Nuri Erdem, Yigit Kultur, Mehmet Aydogan.

**Writing – original draft:** Mehmet Nuri Erdem, Yigit Kultur, Abdulhalim Akar, Mehmet Aydogan.

**Writing – review & editing:** Mehmet Nuri Erdem, Yigit Kultur, Abdulhalim Akar, Mehmet Aydogan.

Supplemental Digital Content is available for this article (https://links.lww.com/MD/P457).

## Supplementary Material
