# Supplementary material for: Local autograft versus mixture of autograft and allograft combination with posterior instrumentation for adolescent idiopathic scoliosis: A retrospective comparative clinical study
Source: Medicine (Baltimore). 2025 Jul 25;104(30):e42443. doi: 10.1097/MD.0000000000042443 (PMC12303472; doi:10.1097/MD.0000000000042443)
Supplement: Supplementary file 1 [file medi-104-e42443-s001.docx]

**Flow chart of the patients**

Patients who underwent adolescent idiopathic scoliosis surgery (n: 59)

Excluded:

- Resides in another country (n: 4)
- Lost to follow-up (n: 1)

Autograft group (N: 28)

Allograft group (n: 26)

Included patients (n: 54)
